# W-Type Hexaferrite Film-Enabled Magnetic Resonance Engineering for Tailored Upper Stop-Band Suppression in Millimeter-Wave Bandpass Filters

**DOI:** 10.3390/mi17050534

**Published:** 2026-04-27

**Authors:** Hyunwoo Koo, Horim Lee, Kyounghwan Kim, Eiyong Park, Yongjun Kim, Sung-Hoon Hong, Sang-Bok Lee, Sungjoon Lim

**Affiliations:** 1 School of Electrical and Electronic Engineering, Chung-Ang University, Seoul 06974, Republic of Korea; koo1006@cau.ac.kr (H.K.); rntqkdl9@naver.com (E.P.); 2 Composites Research Division, Korea Institute of Materials Science, Changwon 51508, Republic of Korea; horimlee@kims.re.kr; 3 Department of Intelligent Semiconductor Engineering, Chung-Ang University, Seoul 06974, Republic of Korea; kevin9711@cau.ac.kr (K.K.); rladydwnshigh@cau.ac.kr (Y.K.); 4 Superintelligence Creative Research Laboratory, Material and Component Research Division, Electronics and Telecommunications Research Institute (ETRI), Daejeon 34129, Republic of Korea; shong@etri.re.kr

**Keywords:** W-type hexaferrite, millimetre-wave bandpass filter, ferromagnetic resonance, stop-band suppression, split-ring resonator

## Abstract

In this study, we propose a novel approach to enhance upper stop-band attenuation in a split-ring resonator-based bandpass filter by partially inserting W-type hexaferrite films into a strategically placed mechanical hole. The hexaferrite exhibits a substantial increase in magnetic loss tangent in the desired band owing to ferromagnetic resonance, considerably improving attenuation in the upper stop-band while maintaining an acceptable insertion loss in the pass-band. The obtained results indicate that selectively placing the hexaferrite film enhances out-of-band rejection by up to 4 dB, with a slight degradation of 0.84 dB in pass-band insertion loss. Before inserting the hexaferrite film, the bandpass filter exhibited an insertion loss of 1.01 dB at 28 GHz and an attenuation of 20.04 dB at 32 GHz. By contrast, after inserting the hexaferrite film, the bandpass filter exhibited an insertion loss of 1.95 dB at 28 GHz and an attenuation of 24.45 dB at 32 GHz.

## 1. Introduction

Bandpass filters (BPFs) are essential components in modern wireless communication systems, radar frontends, and sensing platforms, in which these filters selectively pass desired frequency bands while suppressing unwanted signals with frequencies outside the pass band. In particular, BPFs are required for millimeter-wave applications to achieve not only low insertion loss within the pass-band, but also high attenuation in the stop-bands to mitigate interference, ensure spectral purity, and improve overall system performance.

Achieving strong out-of-band rejection ensures electromagnetic compatibility and maintains signal integrity in high-frequency systems. Inadequate attenuation in the stop bands can generate unwanted spurious emissions, harmonic distortion, and adjacent channel interference, all of which degrade system performance and violate regulatory spectral masks. These are particularly problematic in densely packed frequency environments such as those involving 5G and satellite communications, in which multiple systems coexist within close spectral proximity. Therefore, improving the stop-band attenuation of BPFs is essential for satisfying system-level specifications, complying with international communication standards, and minimizing crosstalk in multi-channel transceivers.

High-performance BPFs, owing to their high speed and low latency characteristics, are essential for 5G applications at the 28 GHz band, a primary frequency band for 5G millimeter-wave deployment [1,2,3,4]. Filters operating in this regime must be compact and compatible with highly integrated modules and maintain low insertion loss (typically < 2 dB) and sharp frequency selectivity to suppress interference from adjacent channels.

However, filter design for millimeter-wave applications encounters several challenges, including high conductor and dielectric losses and high sensitivity to fabrication tolerances [5,6]. Furthermore, achieving high selectivity often requires complex, high-order designs, necessitating large circuits and high tuning complexity [7,8] while deteriorating insertion loss [7].

As a result, there is a growing need for alternative approaches that can achieve strong stop-band attenuation without sacrificing size, manufacturability, or critical filtering performance such as insertion loss. To meet these demands, investigations into numerous filter technologies have been conducted, including low-temperature co-fired ceramic (LTCC) BPFs [9,10,11,12,13,14,15,16], on-chip BPFs [17,18,19,20,21], substrate integrated waveguide (SIW) BPFs [22,23,24,25,26,27,28], and microelectromechanical systems (MEMS)-based BPFs [29,30,31,32,33]. However, these approaches often encounter inherent trade-offs regarding circuit footprint, fabrication cost, and integration complexity [34,35]. Relying on conventional circuit-based structural modifications inevitably increases the physical size. This highlights the need for a paradigm shift toward material-based solutions that can selectively suppress undesired bands without expanding the circuit footprint. Addressing this research gap, this study proposes a fundamentally different approach by utilizing the engineered magnetic resonance of W-type hexaferrite films to achieve targeted upper stop-band suppression. The key idea involves exploiting ferromagnetic resonance (FMR), a frequency-selective magnetic loss phenomenon, as a design variable within the filter architecture. We embedded the ferrite film into a split-ring resonator (SRR)-based BPF, which exhibits a high electromagnetic-field concentration within the ring structure [36,37,38,39,40,41,42]. The interaction between the concentrated magnetic field and the ferrite film is maximized, considerably attenuating the upper stop band.

W-type hexaferrite (BaMe2Fe16O27, where Me is a transition metal) is categorized as a hexagonal ferrite, distinguished by strong uniaxial anisotropy along the c-axis. This intrinsic anisotropic characteristic yields pronounced FMR and high magnetic losses in the gigahertz frequency range, rendering W-type ferrites attractive candidates for various high-frequency applications [43]. As the resonance frequency of FMR is proportional to the magnitude of the anisotropy field (Ha), tailoring the anisotropy field of W-type ferrites frequency-selective magnetic loss responses. Among the approaches investigated for tunable magnetic anisotropy, substituting transition metals for Fe sites has proven to be the most effective and simple strategy for controlling the anisotropy field. In this study, Ni–Co-substituted Ba-W hexaferrite films were fabricated, and the relative amounts of Ni and Co were adjusted to achieve the targeted FMR behavior at the 5G frequency band. The implementation of the hexaferrite films introduce a new aspect of filter design, enabling the precise adjustment of out-of-band rejection by manipulating the magnetic loss profile, rather than relying solely on structural complexity. The proposed strategy combines the electromagnetic concentration capabilities of SRR structures with the FMR of W-type hexaferrite films, offering a compact, integrable, and customizable solution for application in next-generation millimeter-wave filtering systems, particularly in the 28-GHz band.

To validate this approach, W-type hexaferrite films were partially inserted into strategically placed mechanical holes within the SRR-based BPF. Consequently, the magnetic loss tangent of the hexaferrite increased remarkably within the target frequency range owing to FMR, thus considerably improving upper stop-band attenuation while preserving acceptable pass-band performance. Experimental results revealed that the selective placement of the ferrite film enhanced out-of-band rejection by up to 4 dB, with only a minor increase of 0.84 dB in insertion loss. Specifically, the insertion loss at 28 GHz increased from 1.01 dB to 1.95 dB, whereas attenuation at 32 GHz improved from 20.04 dB to 24.45 dB after inserting the hexaferrite film. These results confirm the practical viability of magnetic-resonance-assisted filter design in achieving application-specific suppression in the millimeter-wave regime.

## 2. Design and Simulation

### 2.1. Split-Ring Resonator-Based BPF

**Figure 1:**
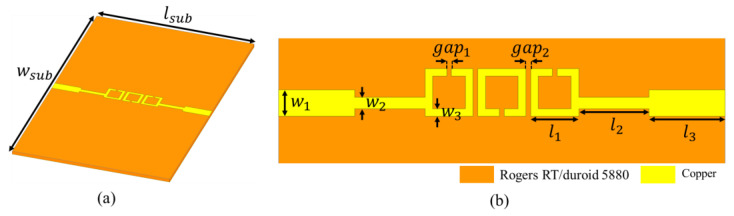
(**a**) Perspective view and (**b**) enlarged top view of the proposed BPF.

**Figure 2:**
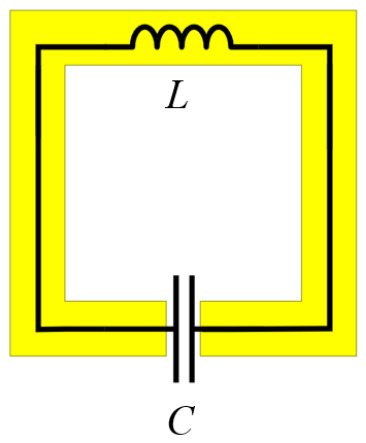
Equivalent LC circuit.

In this paper, we present a third-order SRR-based BPF, operating in the millimeter-wave band as shown in Figure 1. The substrate for the proposed filter is Rogers RT/duroid 5880 with a dielectric constant of 2.2, loss tangent of 0.0009, and thickness of 0.25 mm was used to fabricate the proposed BPF. The thickness of both the top and the bottom metal layers was 0.018 mm. The BPF was designed and simulated using the Ansys high-frequency structure simulator (HFSS) and the simulation applied the finite element method (FEM). Each ring was split, introducing a capacitive element to the structure [36,37,38,39]. An effective inductance (L) and effective capacitance (C) are formed, as shown in Figure 2 [44].

The combination of the inductance and the split-induced capacitance of the ring determined the resonant frequency of the SRR as follows:

(1) $$f=\frac{1}{2\pi \sqrt{\mathrm{LC}}}.$$

**Figure 3:**
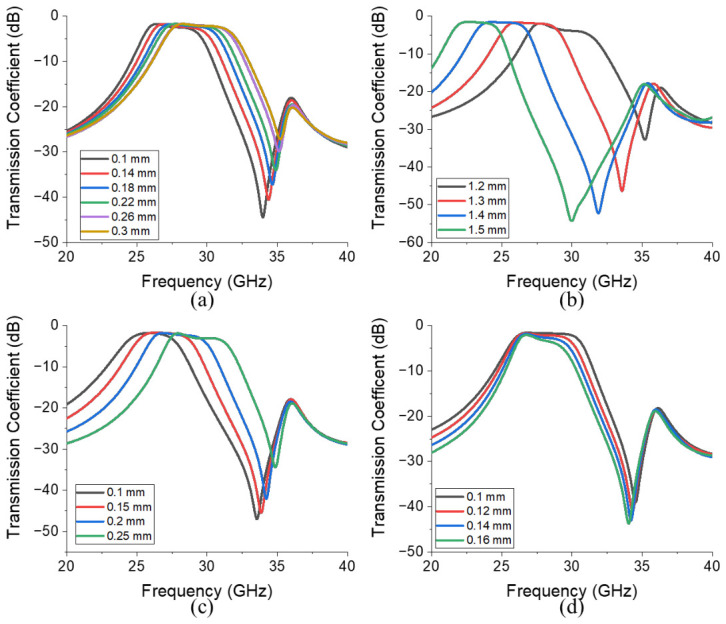
Simulated transmission coefficients obtained by varying the geometric parameters of the BPF illustrated in Figure 1b: (**a**) ${gap}_{1}$ (0.1–0.3 mm), (**b**) $l_{1}$ (1.2–1.5 mm), (**c**) $w_{3}$ (0.1–0.25 mm), and (**d**) ${gap}_{2}$ (0.1–1.6 mm).

The capacitance increased when ${gap}_{1}$ decreased, and the inductance increased when $l_{1}$ increased or $w_{3}$ decreased. This LC resonance behavior is confirmed through simulations, as depicted in Figure 3, which presents the simulated transmission coefficients obtained by varying the geometrical parameters of the filter illustrated in Figure 1b. Figure 3a, Figure 3b and Figure 3c present the transmission coefficients corresponding to the ranges of ${gap}_{1}$ (0.1–0.3 mm), $l_{1}$ (1.2–1.5 mm), and $w_{3}$ (0.1–0.25 mm) values, respectively. The figures reveal that decreases in ${gap}_{1}$ and $w_{3}$ and increases in $l_{1}$ decrease the size of the bandpass region. For example, as ${gap}_{1}$ and $w_{3}$ decreases, the size of the bandpass region decreases from 4.97 GHz (${gap}_{1}$ = 0.3 mm) to 4.17 GHz (${gap}_{1}$ = 0.1 mm), and 4.39 GHz ($w_{3}$ = 0.25 mm) to 3.9 GHz ($w_{3}$ = 0.1 mm), respectively. In addition, as $l_{1}$ increases from 1.2 mm to 1.5 mm, the size of the bandpass region decreases from 4.36 GHz to 3.44 GHz. Moreover, Figure 3d presents the transmission coefficients corresponding to a range of ${gap}_{2}$ values (0.1–0.16 mm), revealing that as ${gap}_{2}$ increases, magnetic coupling decreases [38], which, in turn, increases insertion loss from 1.71 dB (${gap}_{2}$ = 0.1 mm) to 3.26 dB (${gap}_{2}$ = 0.16 mm) at 28 GHz and decreases bandwidth from 5.26 GHz (${gap}_{2}$ = 0.1 mm) to 3.42 GHz (${gap}_{2}$ = 0.16 mm), respectively. Based on these analysis results, the final dimensions of the proposed BPF were determined as follows: $l_{sub}$ = 11.74 mm, $w_{sub}$ = 18.9 mm, $l_{1}$ = 1.26 mm, $l_{2}$ = 1.85 mm, $l_{3}$ = 2 mm, $w_{1}$ = 0.7 mm, $w_{2}$ = 0.3 mm, $w_{3}$ = 0.2 mm, ${gap}_{1}$ = 0.125 mm, and ${gap}_{2}$ = 0.13 mm (Figure 1).

**Figure 4:**
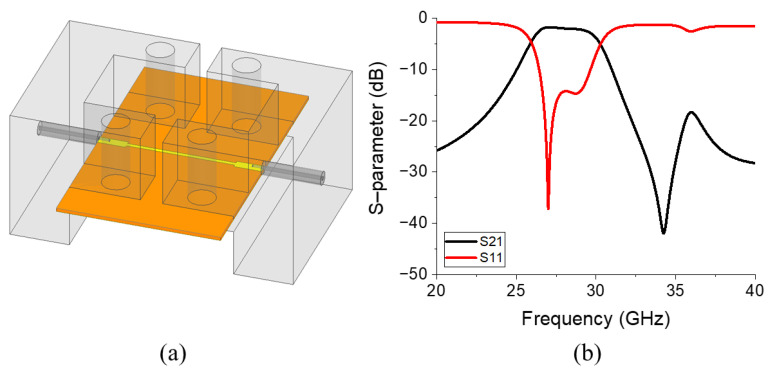
(**a**) Simulation setup of the proposed BPF with a connector and the (**b**) simulated S-parameters. In (**a**), the orange and yellow colors represent the Rogers RT/duroid 5880 substrate and copper, respectively, while the grey parts indicate the connectors.

**Figure 5:**
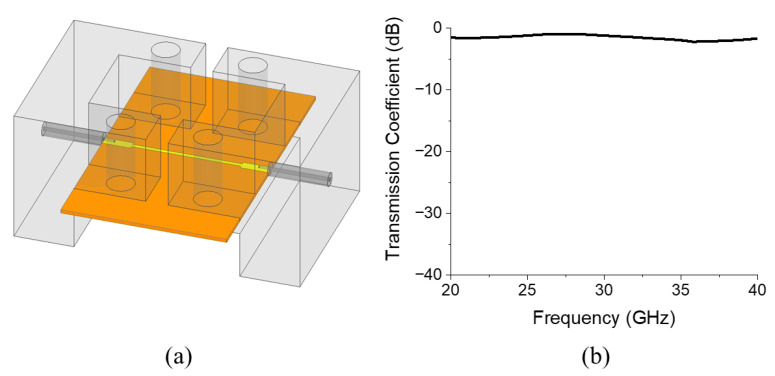
(**a**) Simulation setup of the proposed transmission line with a connector and the (**b**) simulated transmission coefficient. The color representations in (**a**) are identical to those in Figure 4.

The simulation setup of the proposed BPF, including connectors, is displayed in Figure 4a, and the simulated S-parameters are displayed in Figure 4b. To eliminate the insertion loss generated by the transmission line outside the SRR section, including the connectors, a reference transmission line structure is designed, as depicted in Figure 5a. Figure 5b reveals that the insertion loss of this reference transmission line is 0.94 dB at 28 GHz. By subtracting this insertion loss value, the actual insertion loss of the proposed BPF was obtained as 1.02 dB at 28 GHz, and the upper stop band attenuation of the filter was 18.11 dB at 32 GHz.

### 2.2. W-Type Hexaferrite Film

W-type hexaferrite has a general chemical formula of BaMe_2_Fe_16_O_27_ (where Me is a transition metal) and a crystallographic structure consisting of R (BaFe_6_O_11_) and S (2(MeFe_2_O_4_)) blocks stacked in the sequence RSSR^*^S^*^S^*^, where R* and S* blocks are 180° rotated versions of the R and S blocks, respectively. In particular, the R block possesses hexagonal symmetry, which, in turn, imparts the W-type ferrite with a hexagonal crystal structure. This crystallographic characteristic plays a crucial role in determining intrinsically high magnetic anisotropy of W-type ferrites. Such high anisotropy is the origin of FMR, in which the precessional motion of magnetization resonates with the incident electromagnetic wave, generating magnetic losses in the gigahertz frequency range. The resonance frequency of FMR (*f_FMR_*) is proportional to the *H_a_* of the material, as expressed in the following relation:

(2) $$f_{FMR}=\frac{\gamma }{2\pi }H_{a}=1.4gH_{a},$$

where γ represents the gyromagnetic ratio and g is the Lande g-factor [45]. The W-type ferrite must exhibit low magnetic losses within the pass-band while providing high magnetic losses in the upper stop-band for application in BPFs. Therefore, the frequency response of the complex permeability, governed by the FMR, should be carefully tailored by appropriately designing the anisotropy field. The anisotropy field of a W-type ferrite is influenced by several factors, including (1) shape anisotropy, (2) lattice parameters, and (3) Fe sublattice occupancy within the crystal structure. Consequently, precise compositional and processing control is required to achieve the desired performance.

In addition to magnetic losses, a low dielectric loss must be maintained across the operating frequency range to preserve the low insertion loss in the pass band. As W-type ferrites are insulating oxides, they generally exhibit low dielectric losses; however, the coexistence of Fe^2+^ and Fe^3+^ generates higher dielectric losses than M-type ferrites, which are solely composed of Fe^3+^ [46]. Therefore, to minimize dielectric losses, compositional design strategies involving transition-metal substitution must be accompanied by optimized calcination conditions that suppress Fe^2+^ formation.

Ni^2+^-substituted Ba-W ferrite was selected as the candidate material, and Co^2+^ was partially substituted for Ni^2+^ to tailor the FMR frequency to simultaneously achieve (1) high magnetic loss in the upper stop band owing to FMR and (2) low dielectric loss across the entire band [47]. Previous studies have demonstrated that BaNi_2_Fe_16_O_27_ exhibits strong FMR-induced magnetic losses around 40 GHz, while half-substitution of Co^2+^ shifts the resonance frequency downward by nearly 10 GHz. Furthermore, a linear correlation was identified between *f_FMR_* and the Co^2+^ substitution content, as expressed in the following relationship:

(3) $$f_{FMR}\left(GHz\right)=37.49-31.76\left(x\right),$$

where (x) represents the substitution composition of Co ions in the BaNi_2−x_Co_x_Fe_16_O_27_ system. Based on this relation and considering the FMR bandwidth of W-type ferrites, the Co substitution level of 0.1 was considered to induce magnetic losses starting at approximately 30 GHz and produce the maximum imaginary permeability near 35 GHz, corresponding to strong magnetic attenuation in the upper stop-band. Accordingly, we selected BaNi_1.9_Co_0.1_Fe_16_O_27_ as the target composition, synthesized the optimized Ba(W) ferrite powder via controlled calcination, and subsequently fabricated the hexaferrite film applied in this study by using this powder.

### 2.3. BPF with the Hexaferrite Film

**Figure 6:**
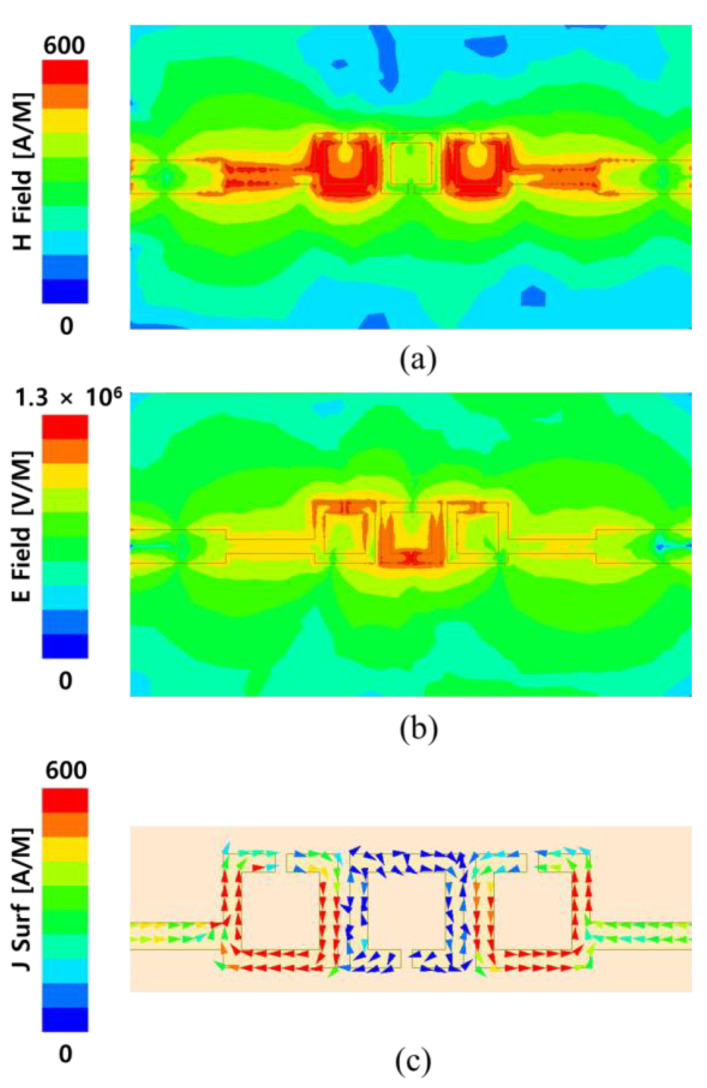
(**a**) Magnitude of the magnetic-field distribution, (**b**) electric-field distribution and (**c**) surface current distributions at 29.9 GHz.

To improve attenuation in the upper stop band, the proposed BPF was first analyzed near the beginning of the stop-band region. Based on the 3-dB cut-off frequency of 30.6 GHz, the electromagnetic-field distribution was examined at 29.9 GHz, which is slightly below the cut-off. Figure 6a indicates that the magnetic field is concentrated in the first and third rings, whereas the electric field is primarily concentrated in the second ring, as depicted in Figure 6b. Furthermore, the surface current analysis shown in Figure 6c indicates a circular current flow, inducing a strong magnetic field inside the ring structure [35].

**Figure 7:**
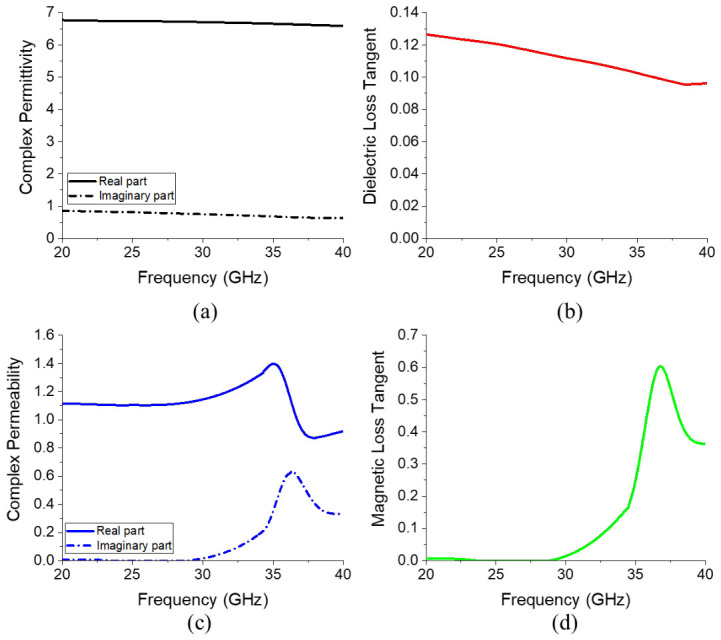
(**a**) Complex permittivity, (**b**) dielectric loss tangent, (**c**) complex permeability, and (**d**) magnetic loss tangent of the ferrite film.

Before inserting the ferrite film at the location where the magnetic field is concentrated, the electromagnetic characteristics of the ferrite film are defined, as shown in Figure 7. To enhance attenuation in the upper stop band, the magnetic loss tangent is configured to begin increasing around 30 GHz, as illustrated in Figure 7d. The frequency-dependent electromagnetic properties of the ferrite film were modeled and assigned in Ansys HFSS using the frequency-dependent material setup.

**Figure 8:**
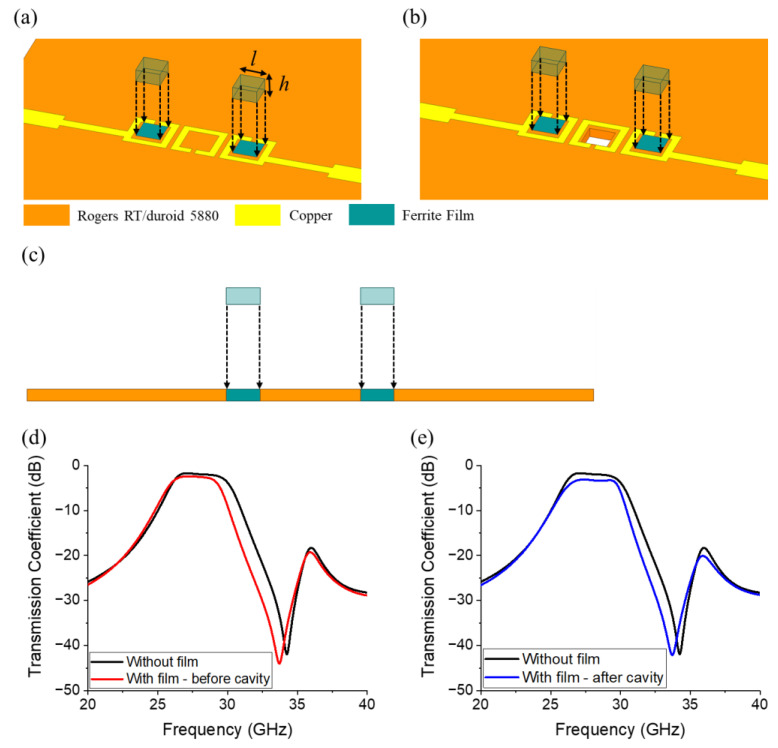
(**a**) Ferrite films inserted into the first and third rings, (**b**) modified structure with an air cavity formed in the concentrated electric-field region, and (**c**) side views of the ferrite films inserted into the first and third rings. Simulated transmission coefficients corresponding to the structure shown in (**d**,**e**).

In the first attempt, ferrite films with lateral dimensions of 0.7 mm and a height of 0.25 mm are inserted into the first and third rings, as displayed in Figure 8a, thereby reducing the bandwidth, as shown in Figure 8c. To address this issue, we refer to the electric-field distribution displayed in Figure 6b and form an air cavity in the region where the electric field is concentrated, as illustrated in Figure 8b. This modification effectively lowered the relative permittivity in the region influenced by the electric field, thereby mitigating the bandwidth reduction. Therefore, the reduced bandwidth is compensated for and attenuation is improved, as shown in Figure 8d [48]. Based on this analysis, the structural configuration was determined such that the ferrite films can be inserted into the first and third rings, and a mechanically formed air cavity can be included in the second ring. The specific dimensions of the ferrite films, however, were not finalized at this stage. To optimize the performance of the BPF, i.e., to maximize stop-band attenuation while minimizing the impact on pass-band insertion loss, a parametric study was subsequently conducted to determine the optimal lateral and vertical dimensions of the ferrite films. In addition to the physical dimensions, the electromagnetic properties of the ferrite film were only partially defined at this stage. These frequency-dependent characteristics require additional analysis and optimization to ensure that the desired filtering performance is attained.

**Figure 9:**
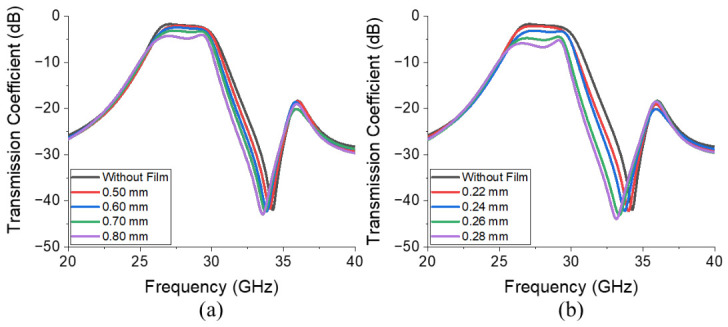
Simulated transmission coefficients corresponding to variations in the geometrical parameters of the ferrite film illustrated in Figure 8a: (**a**) l (0.5–0.8 mm) and (**b**) h (0.22–0.28 mm).

A parametric study is conducted to determine the physical dimensions, l and h, of the ferrite film shown in Figure 8a. Figure 9a presents the transmission coefficients across a range of l values (0.5–0.8 mm). It reveals that as *l* increases from 0.5 mm to 0.8 mm, insertion loss and attenuation in the upper stop band increase from 1.17 dB to 3.19 dB at 28 GHz and from 20.61 dB to 27.32 dB at 32 GHz, respectively. A similar behavior can be observed in Figure 9b, which presents the transmission coefficients corresponding to various *h* values (0.22–0.28 mm). As *h* increases from 0.22 mm to 0.28 mm, insertion loss at 28 GHz increases from 1.27 dB to 5.74 dB, and attenuation at 32 GHz increases from 22.24 dB to 32.66 dB. Because low insertion loss and high attenuation are key to BPFs, the values were selected by considering the trade-offs.

**Figure 10:**
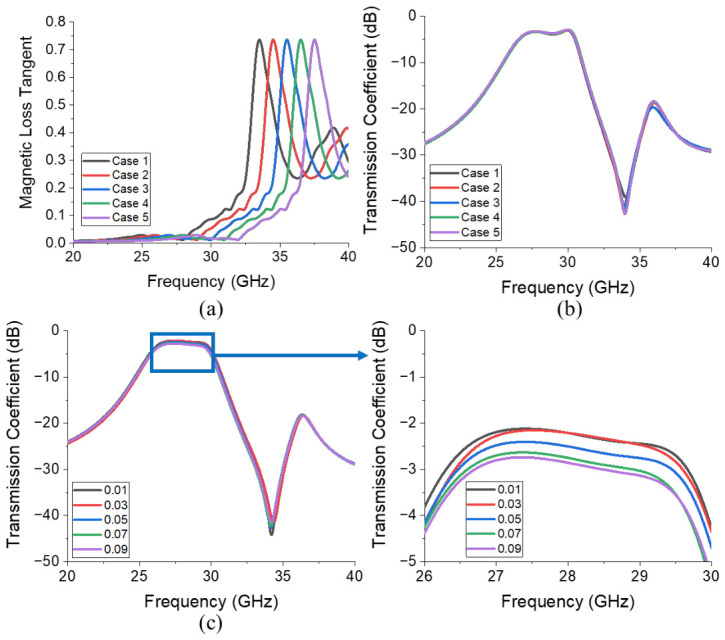
(**a**) Plot of magnetic loss tangent corresponding to different cases with varying magnetic resonance frequencies. (**b**) Simulated transmission coefficients corresponding to each case shown in (**a**,**c**) varying values of dielectric loss tangent.

The analysis for determining the electromagnetic characteristics of the ferrite film was carried out in Figure 10. Figure 10b and Figure 10c present the simulated transmission coefficients corresponding to the different magnetic loss tangent cases illustrated in Figure 10a and different dielectric loss tangent (tan δ) values of the ferrite film, respectively.

Figure 10a reveals that even when the magnetic resonance frequency of the ferrite material varies from 28 to 32 GHz, it negligibly impacts both insertion loss and attenuation, as shown in Figure 10b. However, Figure 10c demonstrates that an increase in the dielectric loss tangent noticeably increases insertion loss from 2.21 dB (tan δ = 0.01) to 2.86 dB (tan δ = 0.09), while minimally affecting attenuation. Therefore, minimizing the dielectric loss tangent is essential.

The optimized parameter dimensions obtained from the geometric parametric analysis of the ferrite film are as follows: l = 0.7 mm and h = 0.25 mm, as per Figure 8a. Furthermore, the electromagnetic analysis presented in Figure 10 reveals that the dielectric loss tangent must be maintained as low as possible, and the magnetic loss tangent should begin to increase near 30 GHz to minimize insertion loss.

**Figure 11:**
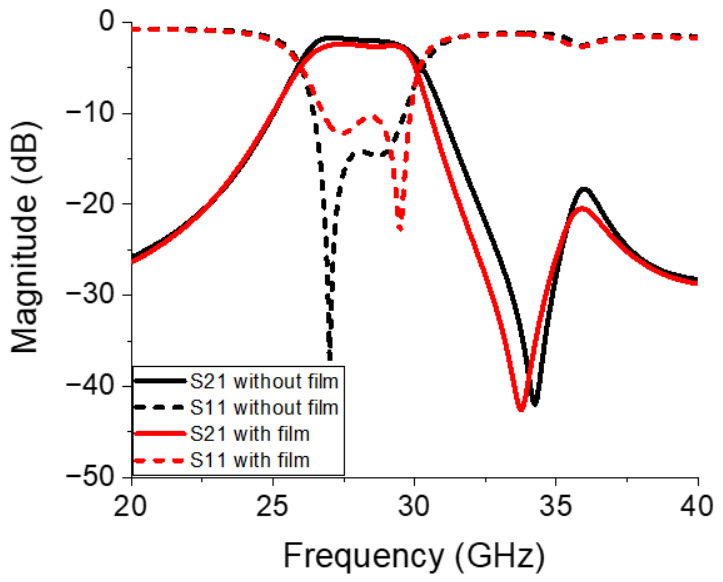
Comparison of the final simulated scattering parameters of the BPF without and with the ferrite film integrated.

Figure 11 compares the final simulation results obtained from the BPF without and with the integration of the ferrite film. Specifically, the filter with the embedded ferrite film exhibited an insertion loss of 1.59 dB at 28 GHz and an attenuation of 23.58 dB at 32 GHz, whereas the insertion loss and attenuation were 1.02 dB and 18.11 dB, respectively, in the design without ferrite film integration. To isolate the performance of the filtering section, these insertion losses are calculated by subtracting the contribution of the reference transmission line, as depicted in Figure 5. These results validate the effectiveness of the proposed magnetic-resonance-assisted filtering approach in terms of achieving excellent stop-band suppression without increasing the complexity or physical footprint of the circuit. The upper stop-band attenuation of the ferrite-integrated BPF improved considerably while maintaining acceptable insertion loss within the pass-band in relation to that obtained using the SRR-only design.

## 3. Fabrication and Measurement

### 3.1. Fabrication

#### 3.1.1. Fabrication of the BPF

**Figure 12:**
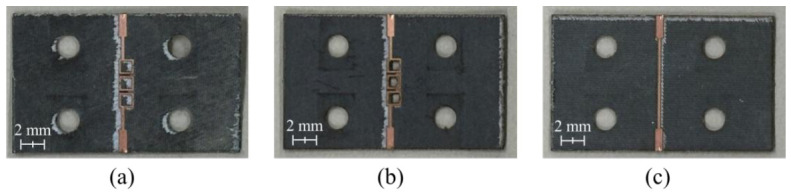
Fabricated proposed BPF (**a**) without and (**b**) with an air cavity and (**c**) a reference transmission line.

The Rogers RT/duroid 5880 substrate(Chandler, AZ, USA) was used to fabricate the proposed BPF. Moreover, the chemical etching process used to prepare conventional printed-circuit boards (PCBs) proved unsuitable for fabricating small features such as the cavity holes with dimensions of 0.7 mm × 0.7 mm. Therefore, a laser etching process developed by LPKF Laser & Electronics for high-precision PCB fabrication was employed. The fabricated BPF is displayed in Figure 12.

#### 3.1.2. Fabrication of the Ferrite Film

The Ba-W ferrite was synthesized using the optimized composition (BaNi_1.9_Co_0.1_Fe_16_O_27_) via a molten-salt-assisted sol–gel method. Briefly, stoichiometric amounts of Ba^2+^, Ni^2+^, Co^2+^, and Fe^3+^ precursors were dissolved in an aqueous solution, to which citric acid was added to initiate the sol–gel reaction. The dried gel powder was subsequently ground and mixed with 50 wt% NaCl, which was the molten-salt medium, followed by calcination at 1250 °C for 3 h. After calcination, any residual NaCl was removed by washing three times with deionized water, yielding single-phase Ba-W hexaferrite powders.

**Figure 13:**
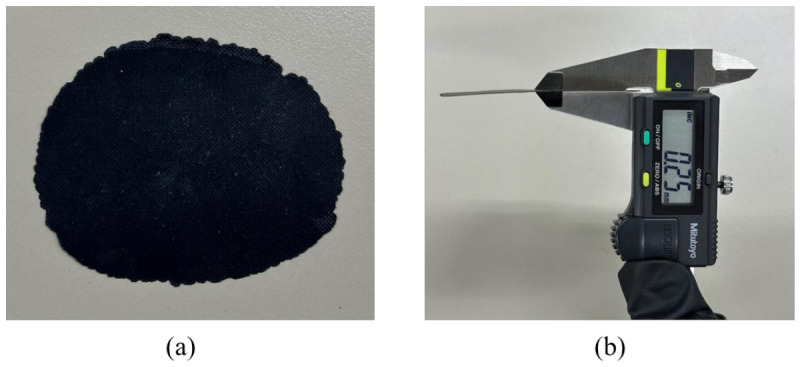
(**a**) Fabricated ferrite film and (**b**) thickness measurement using a vernier caliper.

To fabricate the Ba-W hexaferrite films, the synthesized Ni–Co-substituted Ba-W hexaferrite powders were mixed with a thermoplastic polyurethane (TPU) solution at a weight ratio of 3:7, and the mixture was homogenized using a planetary paste mixer (ARE-310, THINKY, Tokyo, Japan) at 2000 rpm for 3 min, followed by defoaming at 2200 rpm for 2 min. The resulting mixed paste was dried in an oven at 130 °C for 24 h. Finally, the dried mixture was hot-pressed using a high-temperature hydraulic press at 150 °C for 1 h under a uniaxial pressure of 40 tons with a 0.25 mm spacer, yielding a W-type hexaferrite–TPU composite film with the target thickness. The detailed synthesis and film fabrication procedure are reported in our previous work [47]. The fabricated hexaferrite film is presented in Figure 13a, and the thickness of the film, measured using a vernier caliper, is confirmed to be 0.25 mm (Figure 13b).

**Figure 14:**
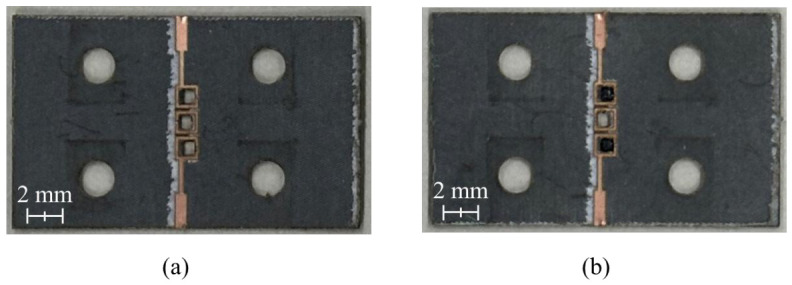
Fabricated proposed BPF with an air cavity (**a**) before and (**b**) after inserting the ferrite films.

Ferrite films with dimensions of 0.7 mm × 0.7 mm are fabricated using a laser cutting machine (C30, Coryart, Seoul, Republic of Korea) and subsequently embedded in the air cavities, as illustrated in Figure 14b.

### 3.2. Measurement Results

#### 3.2.1. Measurement of W-Type Hexaferrite Film

The complex permittivity and permeability values of the ferrite films were measured using a vector network analyzer (N5291A, Keysight, Santa Rosa, CA, USA) via a waveguide and free space measurement system (FS-110, EMLabs, Kobe, Japan) in the K- (18–26.5 GHz) and Ka-band (26.5–40 GHz) frequency ranges. The measurements conducted in the K-band employed a waveguide system, and Keysight K11644A (Santa Rosa, CA, USA) mechanical waveguide calibration kits were used for calibration in each band. The measurements in the Ka-band were conducted using a free-space measurement system, with TRL (thru-reflet-line) calibration conducted by following the protocol provided by the manufacturer.

**Figure 15:**
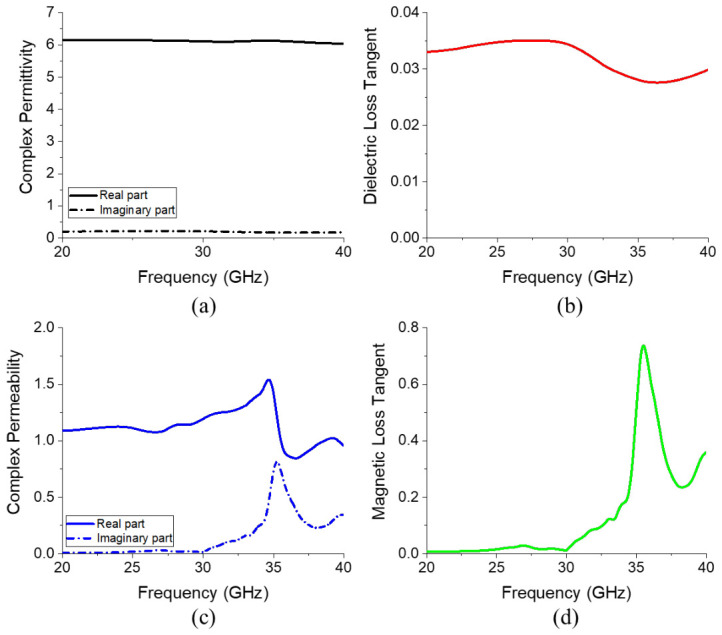
(**a**) Complex permittivity, (**b**) dielectric loss tangent, (**c**) complex permeability, and (**d**) magnetic loss tangent of the final ferrite film.

The measured complex permittivity, dielectric loss tangent, complex permeability, and magnetic loss tangent are presented in Figure 15. The real part of the permittivity remained nearly constant at ~6.2, and the imaginary part was ~0.2 within the measured frequency range, corresponding to a dielectric loss tangent of ~0.03 with minimal variation across this frequency range. In contrast, the complex permeability increased sharply above 30 GHz owing to FMR, reaching a maximum at ~35.5 GHz, at which the peak magnetic loss tangent was 0.736. Compared to the virtual material parameters used in the earlier simulations, the fabricated ferrite films yielded consistently lower dielectric losses across the entire frequency band while exhibiting higher magnetic losses in the upper stop-band.

Furthermore, the onset frequency and spectral profile of the measured permeability closely matched the virtual model, validating the correlation between the FMR frequency and the Co substitution content used in the compositional design. These results confirm that fine-tuning the Co content allows the resonance frequency of the W-type hexaferrite to be precisely controlled.

#### 3.2.2. Measurement of BPF with the Hexaferrite Film

**Figure 16:**
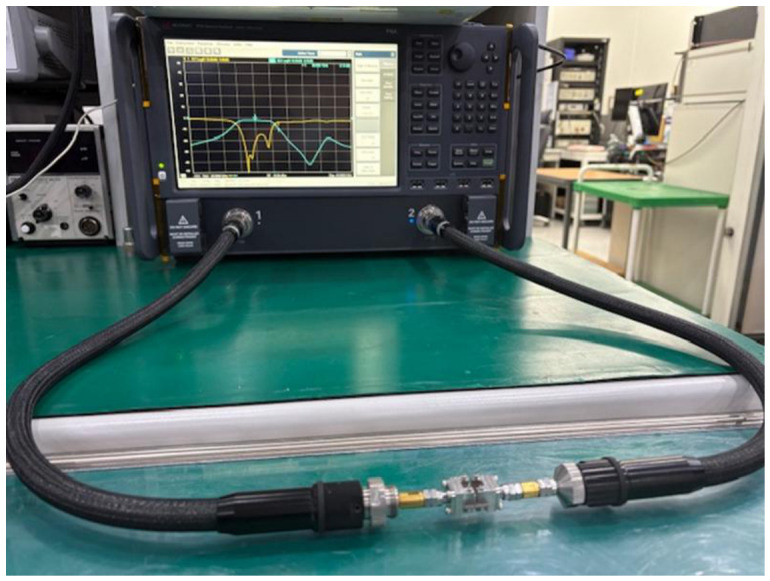
Photograph of the measurement setup.

**Figure 17:**
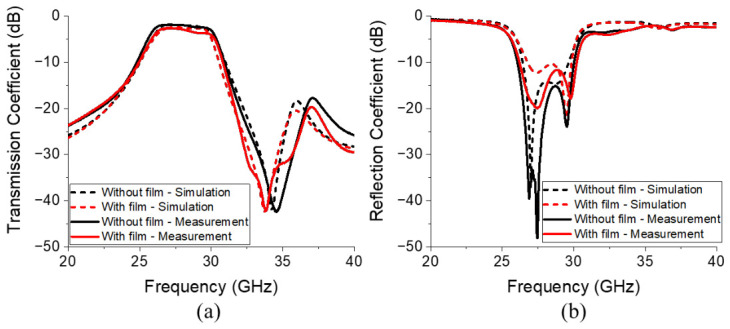
Simulated and measured (**a**) transmission and (**b**) reflection coefficients of the proposed BPF.

**Figure 18:**
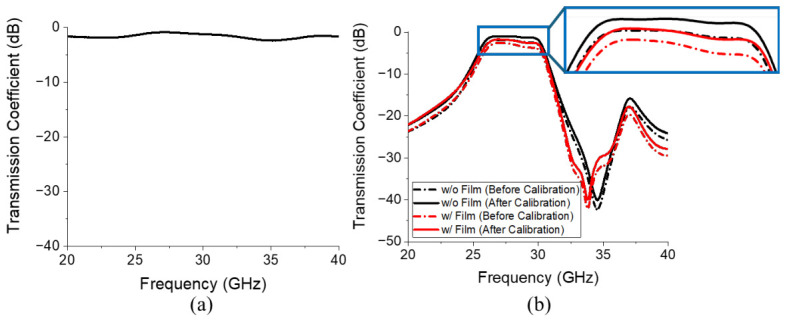
Measured transmission coefficients of the (**a**) reference transmission line and (**b**) comparison of the results obtained before and after calibration. The blue box indicates the magnified region shown on the right.

As illustrated in Figure 16, the S-parameters of the proposed BPF were measured using a performance network analyzer (N5227B, Keysight, Santa Rosa, CA, USA). A full two-port calibration was conducted based on the SOLT (short circuit, open circuit, load, and thru) method using a calibration kit (N4694-60001, Keysight, Santa Rosa, CA, USA) Figure 17 compares the simulated and measured S-parameters of the proposed filter. Specifically, Figure 17a and Figure 17b display the transmission and reflection coefficients, respectively. Figure 17a reveals that the filter with the embedded ferrite film exhibited an insertion loss of 1.95 dB at 28 GHz and an attenuation of 24.45 dB at 32 GHz, whereas the design without ferrite film integration exhibited an insertion loss of 1.01 dB and attenuation of 20.04 dB. These results are obtained after subtracting the insertion loss of 0.93 dB measured at 28 GHz from the reference transmission line, as shown in Figure 18a, and Figure 18b compares the results obtained before and after calibration.

Integrating the ferrite film into the BPF improved attenuation performance by 22%, while maintaining acceptable insertion loss within the pass-band. Table 1 compares the measured and simulated results obtained from the proposed BPF. By comparing the performance before and after the insertion of the magnetic film, a substantial improvement in the upper stop-band attenuation is clearly observed. Specifically, at the target frequency of 32 GHz, the attenuation is enhanced by 5.47 dB in the simulation and 4.41 dB in the measurement. These results quantitatively demonstrate that the W-type hexaferrite film successfully induces tailored magnetic resonance to suppress undesired signals in the upper stop-band while maintaining the pass-band characteristics.

To compare the performance of the proposed filter, a comparison with recent millimeter-wave bandpass filters is summarized in Table 2. Conventional technologies such as LTCC [16], TGV [27], MEMS [29], and multilayer PCB [34] provide high stop-band attenuation. However, they generally suffer from high fabrication complexity and cost due to the requirement of 3D multilayer stacking, intricate glass etching, or sophisticated surface micromachining processes. In contrast, the proposed filter introduces a material-based suppression method utilizing the FMR of the W-type hexaferrite film. This approach achieves a competitive upper stop-band attenuation of 24.45 dB at 32 GHz with a simple planar structure, demonstrating significant advantages in terms of cost-effectiveness, fabrication simplicity, and overall miniaturization.

## 4. Discussion

In this work, a novel approach was proposed for enhancing upper stop-band attenuation in a millimeter-wave BPF, and this method was demonstrated by integrating a W-type hexaferrite film into an SRR-based filter architecture. The proposed method exploits the FMR characteristics of the ferrite material, which were deliberately engineered to exhibit an increasing magnetic loss tangent near 30 GHz while maintaining a low dielectric loss tangent to minimize insertion loss.

Parametric electromagnetic simulations and field-distribution analysis revealed the optimal locations for inserting the ferrite film and forming an air cavity within the SRR structure. A compact square-shaped ferrite film (0.7 mm × 0.7 mm × 0.25 mm) was fabricated and integrated into the regions containing a concentrated magnetic field, while an air cavity was introduced into the region containing a strong electric field to mitigate bandwidth degradation.

Both simulation and measurement results validated the effectiveness of the proposed design strategy. The attenuation offered by the fabricated BPF with the integrated ferrite film improved to 24.45 dB at 32 GHz, representing a 22% enhancement over that offered by the SRR-only design, while maintaining an acceptable insertion loss of 1.95 dB at 28 GHz after calibration. These results confirm that magnetic-resonance-assisted filtering using hexaferrite films can serve as a viable and compact solution for tailored stop-band suppression in millimeter-wave BPFs without increasing circuit complexity and size. The proposed design offers a promising pathway for developing advanced filtering solutions applicable to future 5G and high-frequency communication systems.

## Figures and Tables

**Table 1:** Summary of the simulated and measured results obtained from the proposed BPF.

		Insertion Loss (at 28 GHz)	Attenuation (at 32 GHz)
Simulation result	Without film	1.02 dB	18.11 dB
	With film	1.59 dB	23.58 dB
Measurement result	Without film	1.01 dB	20.04 dB
	With film	1.95 dB	24.45 dB

**Table 2:** Performance comparison of the proposed BPF with millimeter-wave filters.

Ref.	Technology/Structure	$f_{c}$^(a)^ (GHz)	Upper Stop-Band Attenuation (dB)	Fabrication Complexity ^(c)^	Suppression Method
[16]	LTCC/Quad-mode SIW	28	N/A ^(b)^	Complex	Artificial multi-mode cavity
[27]	TGV/HMSIW-DGS	28.3	>20	Complex	Defected ground structure (DGS)
[29]	MEMS/Series & Shunt Bridges	28	N/A	Complex	Tunable electrostatic actuation
[34]	Multilayer PCB/Stacked SIR	28	>24	Medium	Cross-coupling & Harmonic suppression
This work	PCB/SRR	28	24.45	Simple	Ferromagnetic resonance

^(a)^ Center frequency. ^(b)^ N/A: the measured results are not provided in the reference. ^(c)^ Design complexity: complex (including 3D multilayer/intricate glass etching/sophisticated surface), medium (including standard multi-layer PCB stacking/conventional blind/basic mechanical packaging), and simple (including standard single layer-PCB fabrication/simple planar patterning/simple material attachment).

## Data Availability

The data presented in this study are available on request from the corresponding author.
